# Evaluation of the Aptima HBV Quant Assay Compared to Abbott RealTime M2000 HBV Quant Assay and Procleix Ultrio Plus dHBV Assay in Plasma Samples

**DOI:** 10.1128/spectrum.01761-22

**Published:** 2022-07-26

**Authors:** Ying Yan, Le Chang, Huimin Ji, Yang Han, Lunan Wang

**Affiliations:** a National Center for Clinical Laboratories, Institute of Geriatric Medicine, Chinese Academy of Medical Sciences, Beijing Hospital/National Center of Gerontology, Beijing, P. R. China; b Beijing Engineering Research Center of Laboratory Medicine, Beijing Hospital, Beijing, P. R. China; c Department of Infectious Diseases, Peking Union Medical College Hospital, Chinese Academy of Medical Sciences & Peking Union Medical College, Beijing, P. R. China; d Graduate School, Peking Union Medical College, Chinese Academy of Medical Sciences, Beijing, P. R. China; Johns Hopkins Hospital

**Keywords:** HBV DNA, real-time TMA, limit of detection, mutation, dual-target, Aptima HBV Quant assay, Abbott RealTime M2000 HBV Quant assay, Procleix Ultrio Plus dHBV assay

## Abstract

Analytical performance of hepatitis B virus (HBV) DNA quantitative assay is critical for screening infection and initiating and monitoring antiviral treatment. In this study, the limit of detection (LoD) and linearity of Aptima HBV Quant assay were evaluated, and analytical performance was compared with that of the Abbott RealTime M2000 HBV Quant assay and the Procleix Ultrio Plus dHBV assay in plasma samples. The LoDs for genotypes B, C, and D plasma samples were 2.139 (1.531, 4.520), 3.120 (2.140, 7.373), and 3.330 (2.589, 4.907) IU/mL, respectively. The *R*^2^ value fitted by linear regression of serially diluted samples less than 2,000 IU/mL was above 0.9. There was no difference in positive rate between Aptima and Abbott or between Aptima and Procleix. Quantitative results of Aptima and Abbott showed good correlation with an *r* of >0.9 using Spearman analysis, while the quantitative results of Aptima were slightly lower than those of Abbott. Usual mutations in the HBV S region had no impact on Aptima assay. This study showed that Aptima is a dual-targeted transcription-mediated amplification (TMA) assay suitable for HBV DNA detection in clinical practice, with quantitative performance comparable to that of the Abbott RealTime M2000 HBV Quant assay and qualitative performance comparable to that of the Procleix Ultrio Plus dHBV assay.

**IMPORTANCE** The Aptima HBV Quant assay (Hologic Inc., San Diego, CA, USA) is a dual-target real-time transcription-mediated amplification (RT-TMA) assay. This study aims to evaluate whether this assay is suitable for HBV DNA detection. As a result, the assay showed high sensitivity with LoDs below 3.5 IU/mL. The amplification efficiency of Aptima for samples below 2,000 IU/mL is adequate for clinical practice, with an *R*^2^ of >0.9 fitted by linear regression. Usual mutations in the HBV S region did not affect the performance of Aptima. Moreover, its performance was comparable to the widely used Abbott RealTime M2000 HBV Quant assay for detecting HBV DNA in plasma specimens. Although not indicated for use as a diagnostic or blood screening assay, the Aptima HBV Quant assay demonstrated comparable qualitative performance to the Procleix Ultrio Plus dHBV system.

## INTRODUCTION

Overall, the World Health Organization (WHO) estimates that about 257 million people are living with chronic hepatitis B virus (HBV) infection, a major cause of chronic hepatitis B (CHB), cirrhosis, and hepatocellular carcinoma (HCC) worldwide (1). Annually, hepatitis B results in 887,000 deaths (2). HBV infection is still a serious global public health problem.

Chronic HBV infection is a dynamic process reflecting the interaction between HBV replication and the host immune response. In clinical practice, quantification of HBV DNA is the most commonly used indicator to reflect the activity of HBV replication, to determine the phase of infection, and to monitor the initiating time and effect of antiviral treatment (3). The concentrations of HBV DNA are usually above 20,000 IU/mL in HBeAg-positive individuals and below 2,000 IU/mL in HBeAg-negative persons with normal alanine transaminase (ALT) levels (4, 5).

In most instances, HBsAg and HBV DNA are both positive to identify infection. However, HBV DNA can be detected when HBsAg is negative in certain circumstances, such as the window period of infection or in occult HBV infection (OBI) (6). Specifically, the HBV DNA concentration is usually less than 200 IU/mL in OBI, and a minimal infectious dose of 3 IU can cause transfusion-transmitted HBV infection (7). Thus, the sensitivity and accuracy of HBV DNA detection is necessary for recognizing infection and preventing transmission.

Due to the lack of a proofreading mechanism in the HBV reverse transcriptase and because of high viral replication, HBV mutation rates are 100× higher than those of other DNA viruses and almost similar to RNA viruses (8–10). Although primers and probes are designed to target the highly conserved region of the genome, off-target effect remains a concern for reagent manufacturers and users. It was reported that mutation in the probe region of the Cobas Amplicor assay masked HBV drug resistance (11). To avoid underestimation of HBV DNA levels due to mismatch in the targeting region, Aptima HBV Quant assay uses two pairs of primers and probes targeting both the polymerase and surface gene of HBV DNA.

In this study, we aimed to evaluate the limit of detection (LoD) of this dual-target transcription-mediated amplification (TMA) assay and compare the performance of this assay with the Abbott M2000 HBV Quant assay and Procleix Ultrio Plus dHBV assay.

## RESULTS

### Limit of detection.

A series of serially diluted plasma samples of genotypes B, C, and D were tested, and LoDs were calculated by probit analysis. As seen in Table 1, the LoDs of detecting genotype B, C, and D samples were 2.139 (1.531, 4.520), 3.120 (2.140, 7.373), and 3.330 (2.589, 4.907) IU/mL, respectively.

**TABLE 1:** Limit of detection for genotypes B, C, and D

Viral load (IU/mL)	Positive rate (no./total no. [%])		
	Genotype B	Genotype C	Genotype D
8	NA^*a*^	NA	21/21 (100)
4	21/21 (100)	20/21 (95.2)	20/21 (95.2)
2	19/21 (90.5)	18/21 (85.7)	19/21 (90.5)
1	10/21 (47.6)	10/21 (47.6)	8/21 (38.1)
0.5	9/21 (42.8)	6/21 (28.6)	7/21 (30.0)
0.25	1/21 (4.8)	2/21 (9.5)	9/21 (42.8)
0.125	1/21 (4.8)	3/21 (14.3)	4/21 (19.0)
Probit 95% LoD (IU/mL)	2.139 (1.531, 4.520)	3.120 (2.140, 7.373)	3.330 (2.589, 4.907)

a NA, not available.

### Quantitation of low concentration samples.

**FIG 1:**
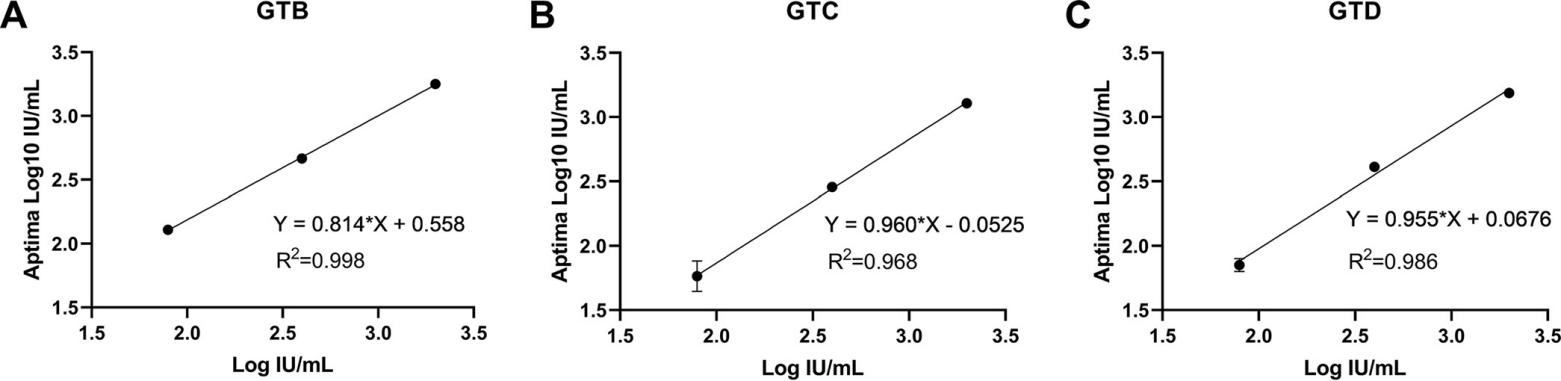
Linearity of Aptima quantitation of genotype B (A), C (B), and D (C) plasma samples.

Samples were serially diluted 5-fold to 2,000, 400, and 80 IU/mL, and three replicates were performed in a single run. Linear regression showed that *R*^2^ was 0.998 for genotype B, 0.968 for genotype C, and 0.986 for genotype D (Fig. 1).

### Comparison strategy.

**FIG 2:**
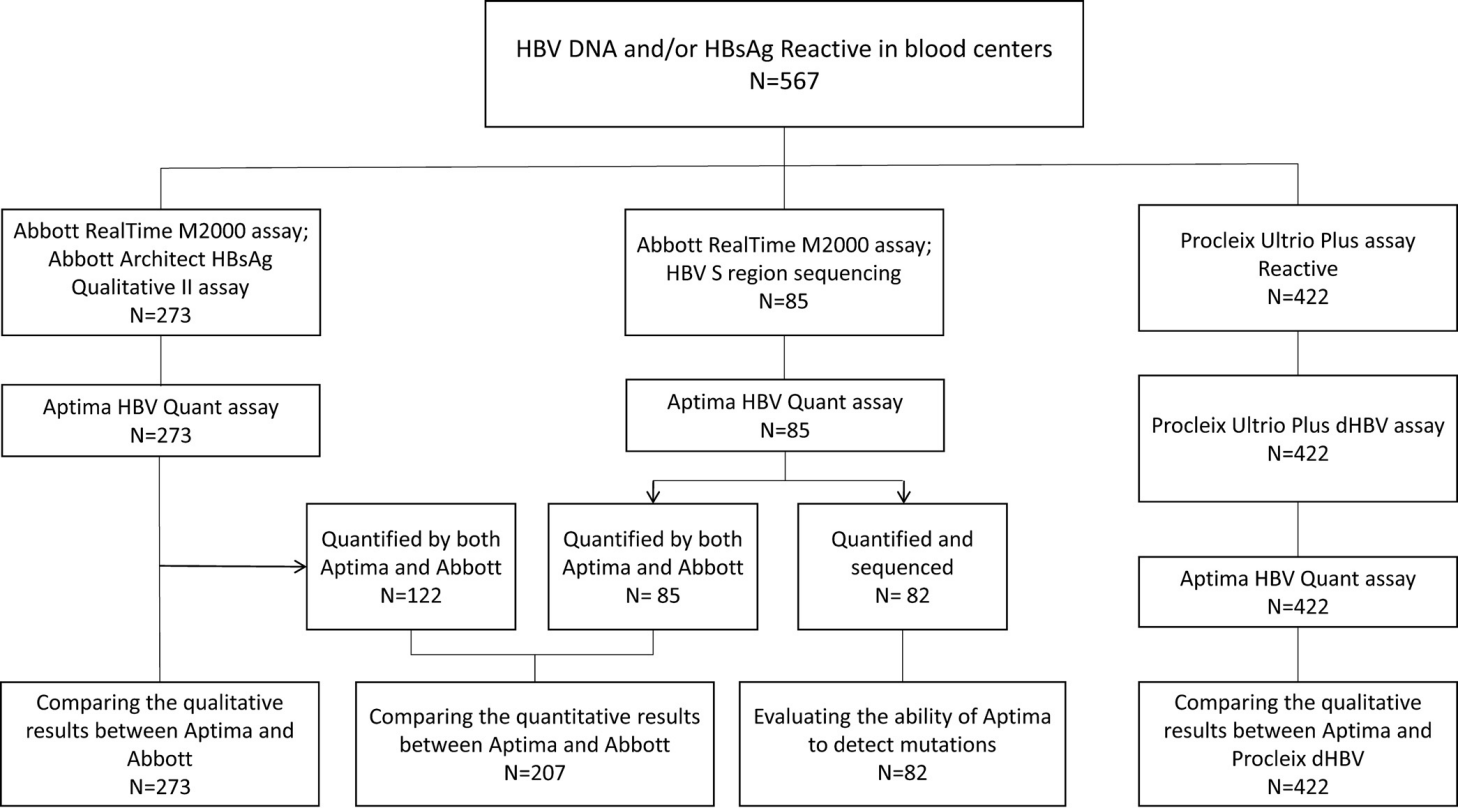
Flow chart of comparing Aptima HBV Quant assay with Abbott RealTime M2000 assay and Procleix Ultrio Plus dHBV assay using plasma samples.

As seen in Fig. 2, among 567 plasma samples that were reactive for HBV DNA and/or HBsAg in blood centers, 273 samples were detected by Abbott RealTime M2000 assay and Abbott Architect HBsAg Qualitative II assay. A total of 85 samples were detected by Abbott M2000, and the HBV S region was sequenced. A total of 422 samples reacted to the Procleix Ultrio Plus assay and were further performed on the Procleix Ultrio Plus dHBV assay. To evaluate the Aptima HBV Quant assay compared to these two HBV DNA assays, all 567 samples were thawed at room temperature and tested using the Aptima HBV Quant assay from August 2021 to December 2021. To compare qualitative ability between Aptima and Abbott in HBsAg-positive and -negative groups, results from 273 samples that were detected by Aptima, Abbott, and Architect HBsAg were analyzed. To compare the quantitative results between Aptima and Abbott, 207 samples that were quantified by both assays were used. A total of 82 samples that were quantified by both assays and successfully sequenced were used to evaluate the ability of Aptima to detect mutations. In the end, results from 422 samples that were detected by both Aptima and Procleix were used to compare the qualitative results between Aptima and Procleix dHBV assays.

### Consistency of qualitative results between Aptima and Abbott assays.

A total of 273 samples were tested for HBsAg using the Architect HBsAg Qualitative II assay and for HBV DNA using Aptima HBV Quant and Abbott M2000 assays. Results were determined following the manufacturer’s instructions and shown in Table 2. To compare the positive rate of these two methods, undetected samples were determined to be negative, while samples detected but not quantified or detected and quantified were determined to be positive. The positive rate was 69.2% for Abbott and 68.9% for Aptima. McNemar’s test showed a *P* value of 1. Positive percent agreement (PPA) (Aptima/Abbott) was 92.1%, negative percent agreement (NPA) (Aptima/Abbott) was 83.3%. Cohen’s kappa statistics showed kappa was 0.751; *P* < 0.001. For 171 HBsAg-reactive samples, positive rate was 94.7% for Abbott and 90.6% for Aptima. The *P* value of McNemar’s test was 0.065. PPA (Aptima/Abbott) was 94.4%, and NPA (Aptima/Abbott) was 77.8%. Cohen’s kappa statistics showed that kappa was 0.528; *P* < 0.001. For those HBsAg-nonreactive samples, positive rate was 26.5% for Abbott and 32.3% for Aptima. McNemar’s test showed that the *P* value was 0.238. PPA (Aptima/Abbott) was 77.8%, and NPA (Aptima/Abbott) was 84.0%. Cohen’s kappa statistics showed that kappa was 0.577; *P* < 0.001. These results showed that there was no difference in positive rate between these two assays, and the qualitative results of these two assays were in good agreement.

**TABLE 2:** Comparing results between Aptima HBV Quant and Abbott RealTime M2000 HBV Quant assays

Aptima HBV Quant group	HBsAg reactive (*n* = 171)				HBsAg nonreactive (*n* = 102)			
	Abbott M2000			Total	Abbott M2000			Total
	Undetected	<15 detected	Quantified		Undetected	<15 detected	Quantified	
Undetected	7	6	3	16	63	3	3	69
<10 detected	2	11	25	38	12	5	11	28
Quantified	0	0	117	117	0	0	5	5
Total	9	17	145	171	75	8	19	102

### Comparison of quantitative results between Aptima and Abbott assays.

**FIG 3:**
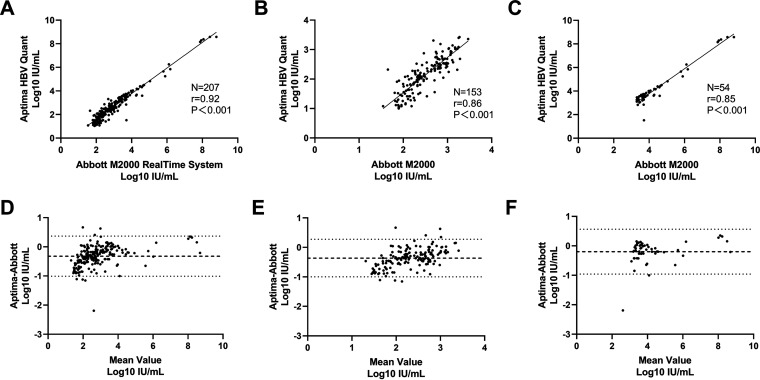
Comparison of quantitative results between Aptima and Abbott assays. Spearman analysis was used to estimate the correlation of all of the samples (A), those below 2,000 IU/mL (B), and those above 2,000 IU/mL (C). Differences were analyzed by Bland-Altman plot (D to F).

A total of 207 samples were quantified by both assays. To investigate the quantitative agreement between Aptima and Abbott, the log_10_ IU/mL concentrations of 207 samples were plotted, and Spearman analysis showed a strong correlation of the quantitative results (*r* = 0.92; *P*<0.001) (Fig. 3A). The differences in log_10_ IU/mL concentrations were plotted against the average values to generate a Bland-Altman plot. This analysis revealed a bias of −0.321 log_10_ IU/mL (Aptima-Abbott) and 95% limits of agreement of −1.008 to 0.367 (Fig. 3D). According to the quantitative results of the Abbott assay, samples were further divided into two groups with 2,000 IU/mL as the boundary. *R* was 0.86 and 0.85, respectively (Fig. 3B and C). Bland-Altman analysis showed a bias of −0.364 (−1.000, 0.276) log_10_ IU/mL for samples <2,000 IU/mL and −0.197 (−0.958, 0.564) log_10_ IU/mL for samples >2,000 IU/mL.

**FIG 4:**
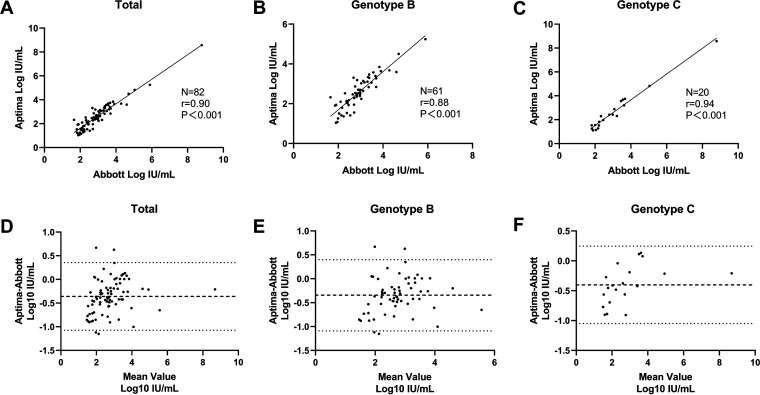
Comparison of quantitative results of mutation panel between Aptima and Abbott assays. Spearman analysis was used for estimation of the correlation of all of the samples (A), genotype B samples (B), and genotype C samples (C). Differences were analyzed by Bland-Altman plot (D to F).

Furthermore, to evaluate whether mutations affect the detection capability of assays, 82 samples with sequencing results for the HBV S region, which were also quantified by both assays, were taken into analysis. Genotype, serotype, and mutations in the HBV S region are listed in Table 3. For all of the samples, Spearman analysis showed a strong correlation of the quantitative results (*r* = 0.90; *P* < 0.001) (Fig. 4A). The Bland-Altman plot revealed a bias of −0.359 (−1.073, 0.355) log_10_ IU/mL (Aptima-Abbott) (Fig. 4D). The *r* value was 0.88 for genotype B and 0.94 for genotype C, while bias was −0.343 (−1.090, 0.399) and −0.402 (−1.050, 0.245) log_10_ IU/mL, respectively. Over 95% of the results fell within the 95% limit of agreement.

**TABLE 3:** Information from the mutation panel

Genotype	Sample no.	Serotype(s)	Mutations
B	61	adw, ayw	Q101H/R, M103I, P105R, L109I/V, I110L, S114A, K122R, T126A, P127T, G130R, M133L, F134I, G145A/K, W156L, F158S, Y161F/S, E164G, V168A, V177A, P178R, V190A, M198I
C	20	adr	Y100F, Q101R/K, K122R, I126T, P127T/A, M133T, T140S, F158S, A159V/G, A166V, R169H, S174N, V177A, V184A, V194A
D	1	ayw	K122R, F134Y, A159G, V168A

### Consistency of qualitative results between Aptima and Procleix Ultrio Plus dHBV assays.

**FIG 5:**
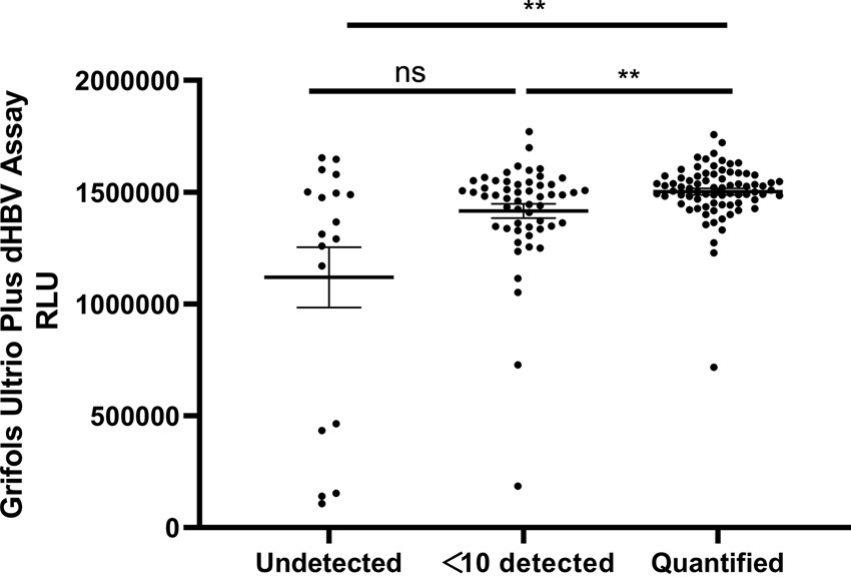
Relative light unit of Procleix Ultrio Plus dHBV assay between three groups divided by the results of Aptima HBV Quant assay.

As assays based on TMA methods, the consistency was analyzed between the Aptima and Procleix Ultrio Plus dHBV system. A total of 442 samples were reactive for Grifols Procleix Ultrio Plus assay. All of the samples were retested using the Aptima and Procleix Ultrio Plus dHBV assays (Table 4). As seen in Fig. 5, the relative light unit reported by Procleix was gradually increased in the undetected group, <10 detected group, and quantified group.

**TABLE 4:** Comparing results between Aptima HBV Quant and Procleix Ultrio Plus dHBV assays

Aptima HBV Quant	Procleix Ultrio Plus dHBV assay		Total
	Nonreactive	Reactive	
Undetected	115	30	145
<15 detected	28	93	121
Quantified	1	175	176
Total	144	298	442

For all 442 samples, the positive rate was 67.4% for Procleix and 67.2% for Aptima, with a *P* value of 1 examined by McNemar’s test. PPA (Aptima/Procleix) was 89.9%, and NPA (Aptima/Procleix) was 79.9%. Cohen’s kappa statistics showed kappa was 0.697; *P* < 0.001. These results suggested that there was no difference in positive rate between these two assays, and the qualitative results of these two assays were in good agreement.

## DISCUSSION

China is an intermediate area where HBV infection is endemic (12, 13). Currently, the weighted prevalence of HBsAg is 5 to 6%, with an estimated 70 million HBsAg carriers (12, 14). Based on sequence diversity, HBV is divided into nine genotypes A through I and one putative genotype J (15). In China, genotypes B and C are mainly prevalent, with a small percentage of genotypes A and D (16). Thus, we evaluated the LoDs of Aptima reagent for detecting genotypes B, C, and D in plasma samples. As stated in the manufacturer’s instructions for use, the LoD is 3 IU/mL, 5.32 IU/mL, and 4.61 IU/mL for these three genotypes, respectively. In this study, the Aptima HBV Quant assay has excellent analytical sensitivity, with estimated lower LoDs in keeping with the manufacturer’s stated values, which are also lower than those of the Abbott M2000 assay.

*R*^2^ fitted by linear regression was above 0.9 when detecting serially diluted samples less than 2,000 IU/mL. Consistently, linearity of Aptima HBV demonstrated excellent correlation with Cobas AmpliPrep/Cobas TaqMan HBV test v2.0 when testing dilution series of three clinical samples of genotypes B, C, and D (17). Both data sets provide evidence that the amplification efficiency of Aptima for low concentration samples is adequate for clinical practice.

HBsAg are markers for active HBV replication. Many studies found that HBV DNA concentration in HBsAg-negative individuals is usually less than 200 IU/mL or can only be intermittently detected (6, 7), which is a challenge for HBV DNA detection reagents. Plasma samples used in this study were from blood donors, a health-conscious population. Most of them do not know they are infected with HBV. Of the 273 samples used to compare Abbott RealTime M2000 HBV Quant assay and Aptima HBV Quant assay, 37.4% (102/273) were HBsAg negative. In the HBsAg-negative group, the positive rate was only 26.5% for Abbott and 32.3% for Aptima, and the PPA (Aptima/Abbott) was 77.8%. In the HBsAg-positive group, positive rate and PPA were all above 90%. Thus, HBV replication is inactive, and HBV DNA levels are relatively low in many samples, which results in a low positive rate and PPA.

Both as quantitative assays, the clinical performances of Aptima and Abbott were well correlated, with an *r* greater than 0.9 for Spearman analysis, and most of the samples fell within the 95% limit of agreement in the Bland-Altman analysis. We found a bias of −0.321 (−1.008, 0.367) log_10_ IU/mL between these two assays. When classified with sample concentrations, the bias was smaller in high-concentration groups (−0.197 log_10_ IU/mL) and became larger as the concentration decreased. Although the procedures of sample transportation, storage, and detection were well controlled, HBV particles degrade with freeze-thaw cycles and prolonged storage time, especially for the low-concentration samples. It was reported that serum HBV DNA levels with baseline concentrations of 7 pg/mL and 55 pg/mL (about 3.5E5 IU/mL and 2.75E6 IU/mL) decreased by over 20% after six freeze-thaw cycles, while samples with higher concentrations only slightly decreased (18). In addition, there was a significant decline in the HBV DNA load after storage at −70°C for up to 4 years (19). To ensure sample diversity, specimens in this study were collected from multiple blood centers across the country and had undergone an average of three freeze-thaw cycles and a 4 year storage at −70°C before Aptima testing. In addition, 30% of samples were below 200 IU/mL reported by Abbott. This might explain the lower quantitation of Aptima and the opposite trends of bias and sample concentration. As reported before, the bias was −0.195 log_10_ IU/mL between the Aptima and CAP/CTM HBV 2.0 assays (17). When comparing with Roche Cobas TaqMan HBV test (HPS/CTM) and Xpert HBV viral load assay, the bias was 0.09 and −0.10 log_10_ IU/mL, respectively (20, 21). Combining our results with all of these comparative studies, we conclude that Aptima has a good performance comparable to that of other widely used HBV quantitative assays.

TMA reaction is an isothermal amplification method that produces single-stranded RNA amplicons from RNA target molecules (22). Using fluorescent tagged probes, it produces 100 to 1,000 copies per cycle rather than the two copies produced by PCR (23). Although TMA has been used for quantification of different pathogens, it is used much less for HBV than the PCR method. The Procleix Ultrio Plus dHBV system is an excellent TMA assay exclusively used for blood screening. The consistency of qualitative results between these two assays was in great agreement, suggesting that Aptima has a good qualitative capability. It is important to note the Aptima HBV Quant assay is not indicated for use as a diagnostic test or blood screening assay but is indicated for use as an aid in the management of patients with chronic HBV infections undergoing HBV antiviral drug therapy.

Mutations can emerge during HBV replication and may be induced under immune stress and drugs. HBV DNA levels could be underestimated because of the mismatch of primer or probe in commercial kits with only one target (24). Our previous study confirmed that a dual target could compensate for the mismatch problem and reduce the risk of false-negative screening (25). Aptima and Abbott have different primers and probes, so quantitative results for one of the reagents will be greatly reduced when testing samples with mismatch mutations. However, when detecting the mutation panel, the results of these two assays were in good correlation, and most of the results fell in the 95% confidence interval. This suggests that Aptima and Abbott both give good quantitative results with no off-target effects in detecting our mutation panel and are suitable for clinical practice.

In conclusion, we reported the evaluation of Aptima HBV Quant assay performed on the Panther system. This assay has excellent sensitivity with an LoD of <3.5 IU/mL for detecting genotypes B, C, and D in plasma. Its performance was comparable to the widely used Abbott M2000 HBV Quant assay for detecting HBV DNA in clinical plasma specimens. Finally, although not indicated for use as a diagnostic or blood screening assay, in this study, the Aptima HBV Quant assay demonstrated comparable qualitative performance to the Procleix Ultrio Plus dHBV system.

## MATERIALS AND METHODS

### Ethics approval.

This study is approved by the Bioethics Committees of Beijing Hospital. The reference number is 2020BJYYEC-149-01.

### Samples.

A total of 567 plasma samples of blood donors with primary screening reactivity for HBV DNA and/or HBsAg were collected from blood centers in 2017. Samples were then transported to the National Center for Clinical Laboratories (NCCL) on dry ice and stored at −80°C. HBV DNA and HBsAg were retested.

### Hologic Aptima HBV Quant assay.

The Aptima HBV Quant assay (Hologic Inc., San Diego, CA, USA) is a real-time transcription-mediated amplification (RT-TMA) assay. It is a dual-target assay designed to be run on the fully automated, random access Panther system. The assay’s limit of detection (LoD) is 5.58 IU/mL for plasma and 4.29 IU/mL for serum, and its lower limit of quantification (LLoQ) is 10 IU/mL with a dynamic range of 1 to 9 Log IU/mL. The input sample volume is 0.5 mL per test. When HBV DNA is detected but at a level below the LLoQ, the result is reported as “<10 detected.”

### Abbott RealTime M2000 HBV Quant assay.

The Abbott M2000 HBV Quant assay is a quantitative assay (Abbott, Abbott Park, IL, USA) run on the M2000 RealTime system. The assay is suitable for use with both 0.5-mL and 0.2-mL sample input volumes. The input sample volume in this study was 0.2 mL. The LoD is 5.61 (3.62 to 10.94) IU/mL, and the LLoQ is 15 IU/mL with a dynamic range of 1.18 to 9 Log IU/mL. When HBV DNA is detected but at a level below the LLoQ, the result is reported as “<15 detected.”

### Grifols Procleix Ultrio Plus assay.

The Grifols Procleix Ultrio Plus assay (Grifols, Emeryville, CA, USA) is a fully automated qualitative TMA assay for the detection of human immunodeficiency virus 1 (HIV-1) RNA, hepatitis C virus (HCV) RNA, and HBV DNA, which is mainly used for blood screening. This assay works on the Procleix Tigris system. The assay firstly combines HIV-1/HCV/HBV signals without discrimination. Specimens found to be reactive will be run in individual HIV-1, HCV, and HBV discriminatory assays to determine if they are reactive for one or any combination of the three. LoD of Ultrio Plus dHBV assay is 4.1 (3.5 to 4.9) IU/mL. The test uses 0.5 mL of specimen volume per test.

### Limit of detection.

LoD was evaluated in accordance with the EP17-A2 guideline (26) of the Clinical and Laboratory Standards Institute. Briefly, plasma samples of genotypes B, C, and D were serially diluted with 5% bovine serum albumin (BSA) containing 0.1% ProClin 300. Each sample was extracted and tested seven times per day in a single run for 3 consecutive days. Positive rate of each dilution was counted, and LoD was calculated from a probit regression model as the measurand concentration at which measurement results yielded a positive classification with a 95% probability. Result determination was completed following manufacturer’s instructions.

### Statistical analysis.

Statistical analysis was performed on IBM (Armonk, NY) SPSS version 21.0. McNemar’s test was performed to examine the differences of positive rate between two assays. Cohen’s kappa statistics was used to determine the agreement of qualitative results between two assays. Spearman analysis was used to estimate the correlation of quantitative results, while Bland-Altman plot was used to determine the bias of the results. Wilcoxon matched-pairs signed rank test was performed to compare the differences of quantitative results between two groups.
